# Evaluation of Digital Drawing Tests and Paper-and-Pencil Drawing Tests for the Screening of Mild Cognitive Impairment and Dementia: A Systematic Review and Meta-analysis of Diagnostic Studies

**DOI:** 10.1007/s11065-021-09523-2

**Published:** 2021-10-16

**Authors:** Joyce Y. C. Chan, Baker K. K. Bat, Adrian Wong, Tak Kit Chan, Zhaohua Huo, Benjamin H. K. Yip, Timothy C. Y. Kowk, Kelvin K. F. Tsoi

**Affiliations:** 1 Department of Medicine and Therapeutics, The Chinese University of Hong Kong, Hong Kong, China; 2 Stanley Ho Big Data Decision Analytics Research Centre, The Chinese University of Hong Kong, Hong Kong, China; 3 JC School of Public Health and Primary Care, The Chinese University of Hong Kong, Hong Kong, China

**Keywords:** Digital drawing test, Digital CDT, MCI, Dementia

## Abstract

Digital drawing tests have been proposed for cognitive screening over the past decade. However, the diagnostic performance is still to clarify. The objective of this study was to evaluate the diagnostic performance among different types of digital and paper-and-pencil drawing tests in the screening of mild cognitive impairment (MCI) and dementia. Diagnostic studies evaluating digital or paper-and-pencil drawing tests for the screening of MCI or dementia were identified from OVID databases, included Embase, MEDLINE, CINAHL, and PsycINFO. Studies evaluated any type of drawing tests for the screening of MCI or dementia and compared with healthy controls. This study was performed according to PRISMA and the guidelines proposed by the Cochrane Diagnostic Test Accuracy Working Group. A bivariate random-effects model was used to compare the diagnostic performance of these drawing tests and presented with a summary receiver-operating characteristic curve. The primary outcome was the diagnostic performance of clock drawing test (CDT). Other types of drawing tests were the secondary outcomes. A total of 90 studies with 22,567 participants were included. In the screening of MCI, the pooled sensitivity and specificity of the digital CDT was 0.86 (95% CI = 0.75 to 0.92) and 0.92 (95% CI = 0.69 to 0.98), respectively. For the paper-and-pencil CDT, the pooled sensitivity and specificity of brief scoring method was 0.63 (95% CI = 0.49 to 0.75) and 0.77 (95% CI = 0.68 to 0.84), and detailed scoring method was 0.63 (95% CI = 0.56 to 0.71) and 0.72 (95% CI = 0.65 to 0.78). In the screening of dementia, the pooled sensitivity and specificity of the digital CDT was 0.83 (95% CI = 0.72 to 0.90) and 0.87 (95% CI = 0.79 to 0.92). The performances of the digital and paper-and-pencil pentagon drawing tests were comparable in the screening of dementia. The digital CDT demonstrated better diagnostic performance than paper-and-pencil CDT for MCI. Other types of digital drawing tests showed comparable performance with paper-and-pencil formats. Therefore, digital drawing tests can be used as an alternative tool for the screening of MCI and dementia.

**Supplementary Information:**

The online version contains supplementary material available at 10.1007/s11065-021-09523-2.

## Background

There is a growing concern globally for mild cognitive impairment (MCI) and dementia due to the increasing aging population. Dementia is a syndrome in which there is progressive deterioration of cognitive function such as memory, visuospatial abilities, executive functions, and thinking (World Health Organization, 2019). MCI is recognized as the intermediate stage between normal aging and dementia (Petersen et al., 1999; Winblad et al., 2004). Early detection of MCI and dementia by cognitive screening tests can help patients and their family members to receive timely proper dementia-related care and support from health care professionals (Prince et al., 2011)

Drawing tests are quick, easy to use, cognitive screening tests that are commonly used for the screening of MCI and dementia. Drawing tests can be applied easily and can assess different neuropsychological functions, such as visuospatial ability and executive functions (Aprahamian et al., 2009; Ehreke et al., 2010). Deterioration of visuospatial abilities and executive functions are common cognitive symptoms in patients with MCI and dementia (Pal et al., 2016; Traykov et al., 2007). There are different types of drawing tests such as the clock drawing test (CDT) (Shulman et al., 1986; Sunderland et al., 1989), pentagon drawing test (Cormack et al., 2004), and cube drawing test (Ota et al., 2015). These tests can be either used alone or combined in a multi-domain cognitive test, such as Montreal Cognitive Assessment (MoCA) (Nasreddine et al., 2005). The CDT is the most extensively used drawing test (Aprahamian et al., 2009; Ehreke et al., 2010). In the early development of the CDT, it was mainly used to screen visuospatial and visual-constructional disorders associated with lesions in the parietal region of the brain, such as post-stroke dementia (Aprahamian et al., 2009). The usage of CDT is widened nowadays, and the CDT is widely used for the screening of MCI and dementia (Tsoi et al., 2015). There are different scoring methods of the CDT such as the 6-point scoring method (Shulman et al., 1986) and the 10-point scoring method (Sunderland et al., 1989). Studies indicate that the CDT is a good test for the screening of patients with dementia (Aprahamian et al., 2009; Park et al., 2018), but that MCI patient performance is fair and may overlook degenerative processes (Breton et al., 2019; Ehreke et al., 2010; Pinto & Peters, 2009; Tsoi et al., 2017).

Due to the weakness of paper-and-pencil drawing tests and the advancement of technology, digital drawing tests have evolved over the past decade. Digital drawing tests can record and assess drawing characteristics such as total time spent, contour, and drawing methods, which can be considered when discriminating between MCI and dementia (Heymann et al., 2018). Studies showed that on-air movements can enhance the sensitivity of identifying patients with MCI (Garre-Olmo et al., 2017; Müller et al., 2017). The pressure applied when drawing can be another indicator to discriminate elders with MCI and healthy aging (Faundez-Zanuy et al., 2013).

A meta-analysis found that the diagnostic performance of digital cognitive tests and paper-and-pencil cognitive tests are comparable (Chan et al., 2018). However, previous studies seldom compare the diagnostic performance of digital drawing tests and paper-and-pencil drawing tests specifically. Therefore, the objective of this study was to evaluate the diagnostic performance of different types of digital drawing tests and paper-in-pencil drawing tests for the screening of MCI and dementia.

## Methods

The current study was performed according to the standard guidelines for the systematic review of diagnostic studies, including the Preferred Reporting Items for Systematic Reviews and Meta-Analyses (PRISMA) (Moher et al., 2009) and the guidelines proposed by the Cochrane Diagnostic Test Accuracy Working Group (Leeflang et al., 2008; Macaskill et al., 2010). This study is registered as CRD42020166750 in PROSPERO.

### Search Strategy

Literature searches were performed in OVID databases, included Embase, MEDLINE, CINAHL, and PsycINFO. Keywords included “cognitive impairment”, “MCI”, “dementia”, “draw test”, “digital draw”, “Clock drawing test”, “Pentagon test”, “Cube draw”, “Tree draw”, “House draw’ and “Rey-Osterrieth”, “ROCF”, “Spiral” and “infinity loops” (Supplementary Table 1). The search duration was from the earliest available dates in each database to the 31st of March 2020. Diagnostic studies comparing the accuracy of the drawing tests for MCI and dementia were identified from the title and abstract preview of all search records. Literature searches were also extended to the Digital Dissertation Consortium database and WorldCat for identification of unpublished theses or grey literature. Manual searches were extended to the bibliographies of the review articles and studies that were included in this meta-analysis. No language restriction was adopted.

### Inclusion and Exclusion Criteria

Studies were included if they met the following inclusion criteria:

1. The study used any type of drawing tests for the screening of MCI or dementia;
2. The study recruited participants with MCI or dementia in any clinical or community settings and compared them with cognitively healthy controls;
3. Participants with MCI or dementia were confirmed with standardized diagnostic criteria, including the Petersen criterion (Petersen et al., 1999), the report of International Working Group on Mild Cognitive Impairment (Winblad et al., 2004), the recommendations of the National Institute on Aging and the Alzheimer’s Association (Albert et al., 2011), the National Institute of Neurological and Communicative Disorders and Stroke and the Alzheimer’s Disease and Related Disorders Association (NINCDS-ADRDA) (McKhann et al., 1984), any version of the Diagnostic and Statistical Manual of Mental Disorder (DSM) (American Psychiatric Association, 2013), consensus by qualified clinicians using the Clinical Dementia Rating (CDR) (Morris, 1993) or standardized neuropsychological tests. Studies reported cases of very mild dementia, questionable dementia or cognitive impairments with no dementia (CIND), were further studied to confirm whether they included participants with MCI;
4. The diagnostic performance of the drawing tests was summarized in terms of sensitivity and specificity, or data that could be used to derive those values were provided.

Studies were excluded if a study only evaluated different types of MCI or dementia, such as to identify patients with Parkinson’s disease dementia from Alzheimer’s disease.

### Data Extraction

Two investigators (TKC, BKK) independently evaluated the relevance of search results and extracted the data into an Excel spreadsheet. The spreadsheet was used to record the demographic details of included articles, such as the year of publication, the study location, the number of MCI or dementia participants and controls, the mean age of participants, the percentage of male participants, and the diagnostic criteria and cutoff values used to define patients with MCI or dementia. We also recorded the sensitivity and specificity, or true-positive, false-positive, true-negative, and false-negative values, of each drawing test for result analysis. When a study presented different cutoff values to show the performance of a test, only the result from a recommended cutoff value in the article was chosen. When a study recommended more than one cutoff value, the cutoff value presented in the abstract of the article was selected. When there were discrepancies in the study eligibility or data extraction, the third investigator (JYC) would make the definitive decision. The Cohen’s kappa was used to evaluate the inter-rater variability.

### Type of Drawing Tests

The digital drawing tests included the digital CDT, digital pentagon drawing test, digital Rey-Osterrieth complex figure (ROCF), digital tree drawing test, digital house drawing test, and digital spiral test. The paper-and-pencil drawing tests included the CDT, pentagon drawing test, cube drawing test, and ROCF. We further categorized paper-and-pencil CDT into the brief scoring method (i.e. ≤ 9 score), and the detailed scoring method (i.e. > 9 score).

### Risk of Bias and Reporting Quality

The Quality Assessment of Diagnostic Accuracy Studies 2 (QUADAS-2) instrument (Whiting et al., 2011) was used to evaluate the potential risks of bias (ROB). The assessment areas included, 1. selection of patient, 2. execution of the screening tests, 3. execution of the reference standard, and 4. presentation on the patient flow and timing to have the reference standard and index tests. The methodology section of the STARD statement (Standards for Reporting of Diagnostic Accuracy) (Bossuyt et al., 2003) was used to evaluate the study quality. An 8-point scale was designed to evaluate the study quality, which included: 1. a clear definition on study population, 2. adequate details of recruitment of participants, 3. description of sampling of participant selection, 4. description of data collection plan, 5. description of reference standard and its rationale, 6. specifications of the drawing tests, 7. rationales for cutoff values, and 8. methods of calculation of diagnostic performance.

### Outcomes

The primary outcome of this study was the diagnostic performance of the CDT for the screening of MCI and dementia. The secondary outcome was the diagnostic performance of other types of drawing tests.

### Data Synthesis and Statistical Analysis

A bivariate random-effects model was used to combine the overall sensitivity and specificity of each drawing test (Reitsma et al., 2005). Forest plots were used as the graphical presentation for the pooled sensitivity and specificity. A diagnostic odds ratio was used as a single indicator of the test performance across different thresholds of cutoff values (Glas et al., 2003). A hierarchical summary receiver-operating characteristic (HSROC) curve was generated to present the summary estimates of sensitivity and specificity along with corresponding 95% confidence interval (95% CI) and prediction region (Rutter & Gatsonis, 2001). The area under the HSROC curve (AUC) was calculated. The approach of DerSimonian and Laird was applied when Hessian matrix was unstable (DerSimonian & Laird, 1986). Statistical heterogeneity among the trials was assessed by I^2^. The diagnostic performances of digital and paper-and-pencil drawing tests were compared by meta-regression models, with *P* < 0.05 indicates a statistically significant difference. Publication bias was conducted by a regression of diagnostic log odds ratio against 1/sqrt (effective sample size), weighting by effective sample size, with *P* < 0.10 for the slope coefficient indicating significant asymmetry (Deeks et al., 2005). Sensitivity analysis was conducted according to the scoring methods of paper-and-pencil clock drawing tests. The statistical analyses were performed with the Midas procedures in STATA, version 11 (StataCorp) and meta-disc version 1.4.

## Results

### Literature Search and Study Selection

**Fig. 1:**
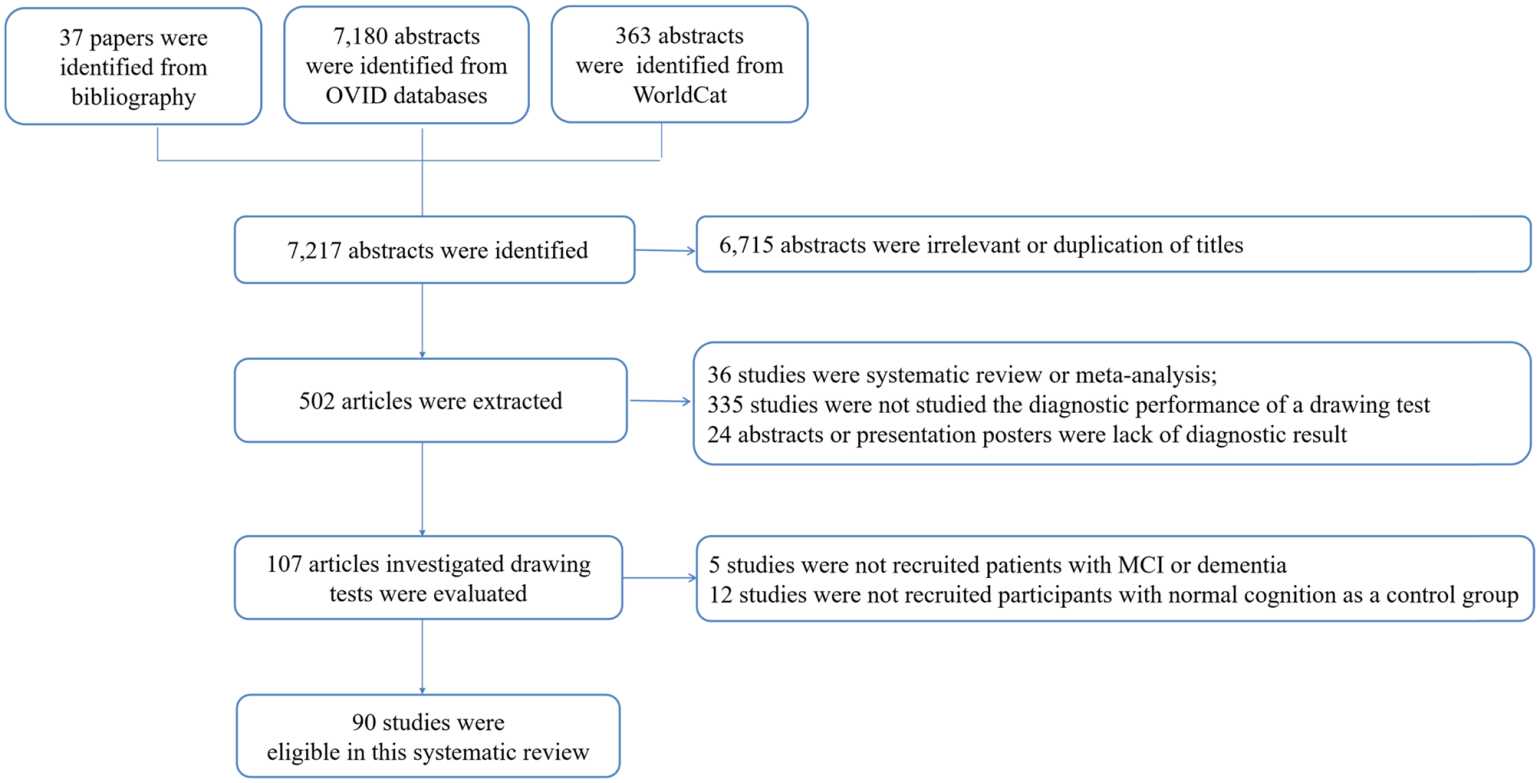
Summary of Literature search

A total of 7,180 abstracts were identified in OVID databases, 37 papers were identified from the bibliography, and 363 abstracts were identified from WorldCat. After excluding the irrelevant or duplication of titles, 502 articles were further evaluated. A total of 412 articles were excluded (Cohen’s Kappa statistics at 85% between the investigators) due to the following reasons: 36 studies were systematic review or meta-analysis, 335 studies were not studied the diagnostic performance of a drawing test, 24 abstracts or presentation posters were lack of diagnostic result, 5 studies were not recruited patients with MCI or dementia, and 12 studies were not recruited participants with normal cognition as a control group. As a result, a total of 90 studies were eligible for this systematic review and meta-analysis (Fig. 1).

### Study Characteristics

A total of 90 studies with 2,810 participants with MCI, 7,751 participants with dementia, and 12,006 controls were included in this systematic review and meta-analysis. The mean age of the participants ranged from 58 to 85 (Supplementary Table 2). The percentage of male participants ranged from 4 to 99%. Seventy-six studies recruited participants with MCI or dementia in an out-patient clinic or the community, whereas all other studies recruited participants in hospital or an old age home. Six studies used digital CDT, while other digital drawing tests included pentagon drawing test (n = 2), tree drawing test (n = 2), ROCF (n = 1), house drawing test (n = 1), and spiral drawing test (n = 1). Paper-and-pencil CDT was the most commonly used drawing test, as 45 studies used the detailed scoring method (i.e. > 9 score), and 35 studies used the brief scoring method (i.e. ≤ 9 score). Other paper-and-pencil drawing tests included the pentagon drawing test (n = 5), cube drawing test (n = 3) and ROCF (n = 1). In the assessment of ROB, nine studies were assessed as high risk of bias, including selection of patients (n = 6), execution of the index test (n = 1), execution of the reference standard (n = 3) and flow and timing (n = 4) (Supplementary Table 3).

### Performance of Digital and Paper-and-pencil CDT in the Screening of MCI

**Fig. 2:**
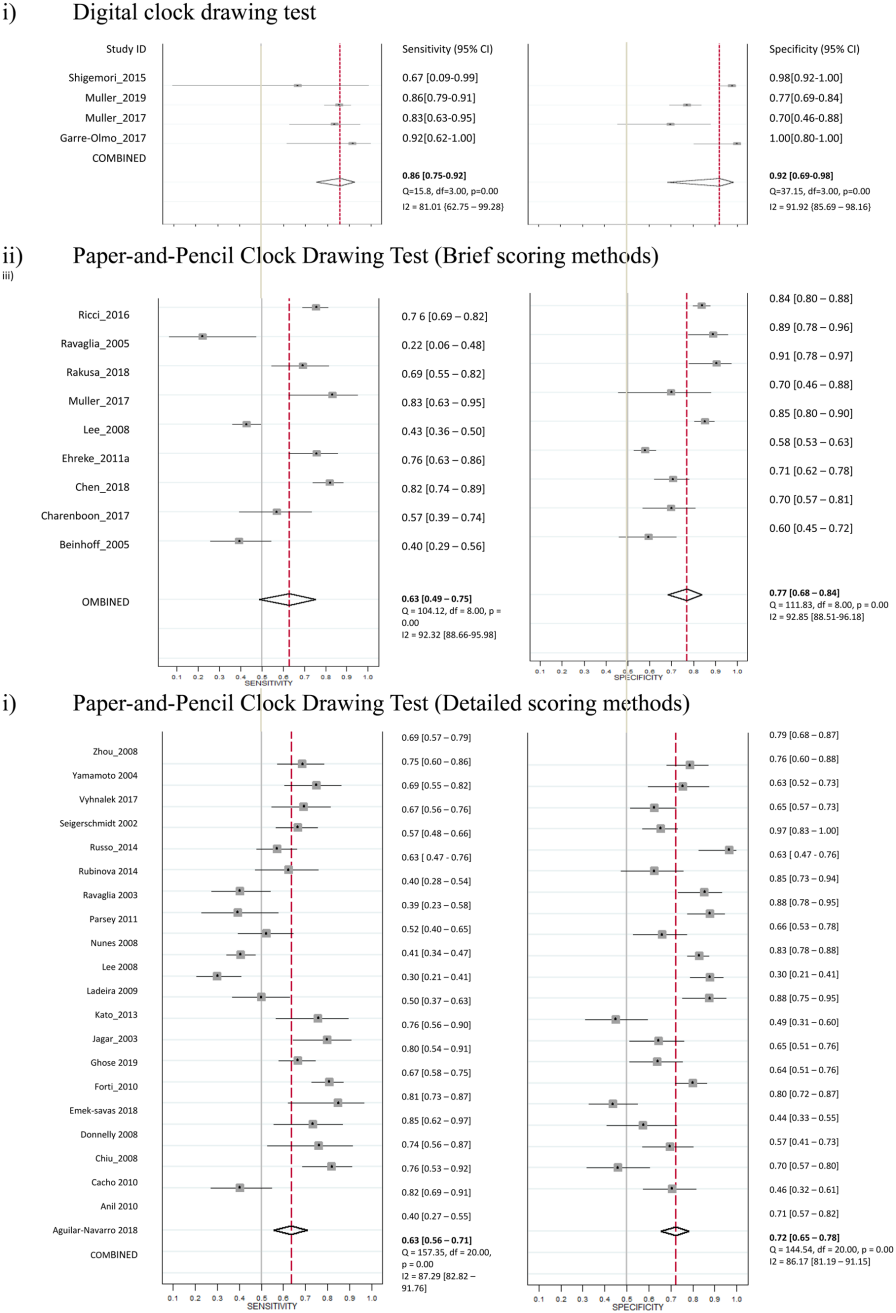
Studies of Digital and Paper-and-Pencil Clock Drawing Tests for the Screening of MCI

Four studies used the digital CDT for the screening of MCI, and a total of 179 participants with MCI and 467 controls were included. The sensitivities ranged from 0.67 to 0.94 and specificities ranged from 0.79 to 0.94 across individual studies. The heterogeneity with I^2^ statistic for sensitivity and specificity were 0.81 and 0.92, respectively. The pooled sensitivity and specificity of four studies using digital CDT were 0.86 (95% CI = 0.75 to 0.92) and 0.92 (95% CI = 0.69 to 0.98), respectively (Table 1a, Fig. 2, Supplementary Table 4). The pooled AUC was 87% (95% CI = 84% to 90%) (Supplementary Fig. 1). A non-significant *P*-value (0.42) for the slope coefficient suggested symmetry in the data and a low likelihood of publication bias (Supplementary Fig. 2). Nine studies used brief scoring method of paper-and-pencil CDT. The heterogeneity with I^2^ statistic for sensitivity and specificity were 0.92 and 0.93, respectively. The pooled sensitivity and specificity were 0.63 (95% CI = 0.49 to 0.75) and 0.77 (95% CI = 0.68 to 0.84), respectively. The pooled AUC was 77% (95% CI = 74% to 81%). Twenty-one studies used detailed scoring method of paper-and-pencil CDT. The heterogeneity with I^2^ statistic for sensitivity and specificity were 0.87 and 0.86, respectively. The pooled sensitivity and specificity were 0.63 (95% CI = 0.56 to 0.71) and 0.72 (95% CI = 0.65 to 0.78), respectively. The pooled AUC was 74% (95% CI = 69% to 77%). The diagnostic performance of digital CDT was significantly better than the brief scoring methods (*P* = 0.02) and detailed scoring methods (*P* < 0.001) of paper-and-pencil CDT in the meta-regression models.

**Table 1:** Sensitivity and Specificity of Digital and Paper-and-Pencil Clock Drawing Tests

**Type of Clock Drawing Tests**	**No. of study**	**Sensitivity** **(95% CI)**	**Specificity** **(95% CI)**	**LR +** **(95% CI)**	**LR-** **(95% CI)**	**DOR** **(95% CI)**	**AUC** **(95% CI)**
**a. MCI**							
Digital CDT	4	0.86 (0.75–0.92)	0.92 (0.69–0.98)	10.6 (2.30–49.0)	0.15 (0.08–0.29)	69.0 (10.6–449)	87% (84%–90%)
Paper-and-pencil CDT –Brief Scoring (≤ 9 points)	9	0.63 (0.49–0.75)	0.77 (0.68–0.84)	2.74 (1.94–3.88)	0.48 (0.34–0.68)	5.71 (3.06–10.7)	77% (74%–81%)
Paper-and-pencil CDT –Detailed Scoring (> 9 points)	21	0.63 (0.56–0.71)	0.72 (0.65–0.78)	2.29 (1.92–2.73)	0.50 (0.43–0.59)	0.54 (3.47–5.93)	74% (69%–77%)
**b. Dementia**							
Digital CDT	6	0.83 (0.72–0.90)	0.87 (0.79–0.92)	6.35 (3.9–10.5)	0.20 (0.12–0.33)	32.2 (13.7–75.9)	92% (89%–94%)
Paper-and-pencil CDT **–**Brief Scoring (≤ 9 points)	30	0.83 (0.77–0.87)	0.80 (0.74–0.85)	4.07 (3.15–5.26)	0.22 (0.16–0.29)	18.7 (12.2–28.7)	88% (85%–91%)
Paper-and-pencil CDT –Detailed Scoring (> 9 points)	35	0.80 (0.76–0.83)	0.81 (0.75–0.86)	4.24 (3.24–5.54)	0.25 (0.21–0.30)	17.1 (12.0–24.5)	87% (84%–90%)

*CDT* Clock Drawing Test, *MCI* Mild Cognitive Impairment, *LR* + Positive likelihood ratio, *LR-* Negative likelihood ratio, *DOR* Diagnostic odds ratio, *AUC* Area under the curve, *CI* Confidence interval

### Performance of Digital and Paper-and-pencil CDT in the Screening of Dementia

**Fig. 3:**
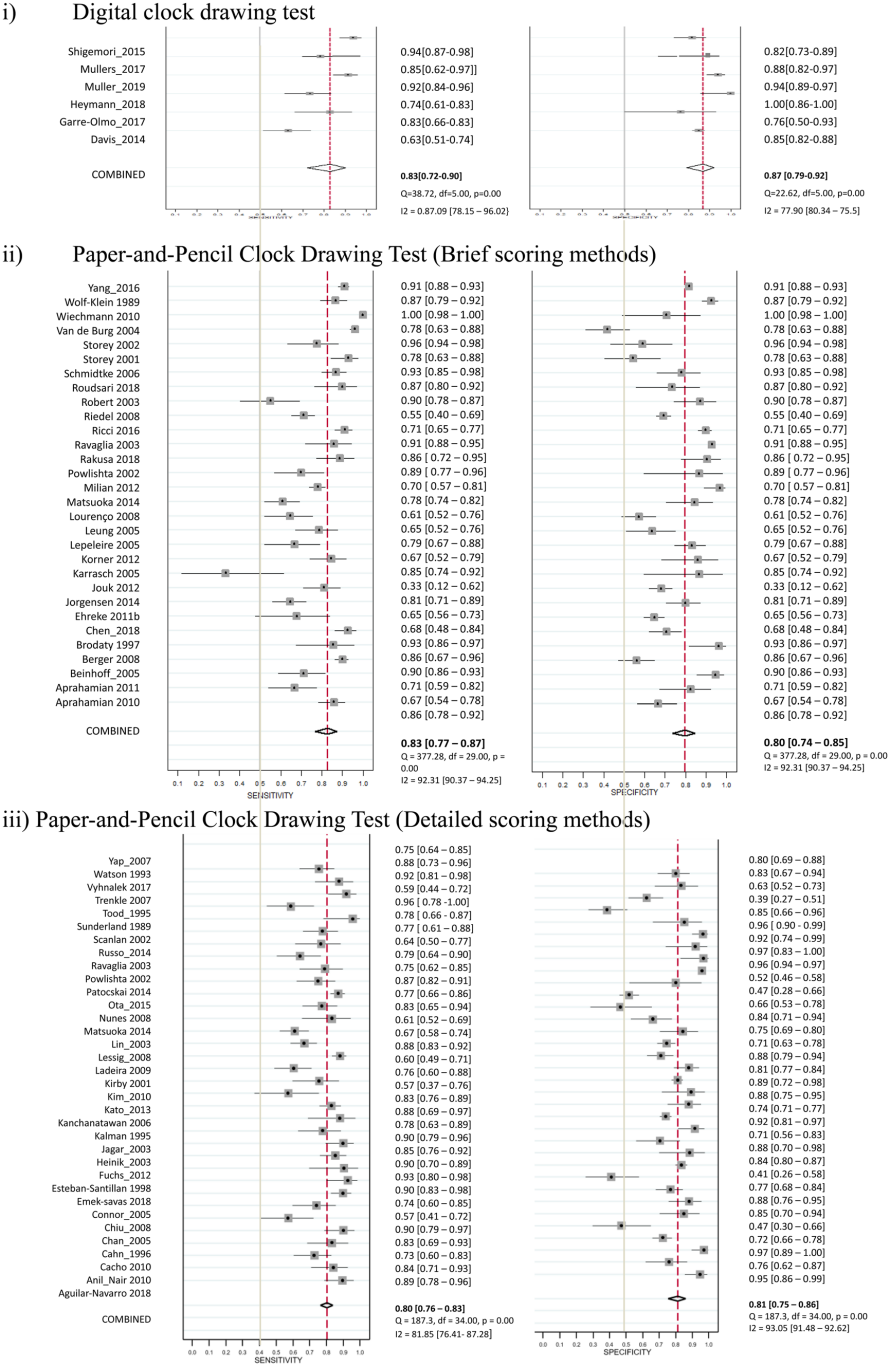
Studies of Digital and Paper-and-Pencil Clock Drawing Tests for the Screening of Dementia

Six studies used the digital CDT for the screening of dementia, and a total of 505 participants with dementia and 915 controls were included. The sensitivities ranged from 0.63 to 0.94 and specificities ranged from 0.77 to 1.00 across individual studies. The heterogeneity with I^2^ statistic for sensitivity and specificity were 0.87 and 0.78, respectively. The pooled sensitivity and specificity with bivariate random-effects model were 0.83 (95% CI = 0.72 to 0.90) and 0.87 (95% CI = 0.79 to 0.92) (Table 1b, Fig. 3). The pooled AUC was 92% (95% CI = 89% to 94%) (Supplementary Fig. 3). A non-significant *P*-value (0.93) for the slope coefficient suggested symmetry in the data and a low likelihood of publication bias. Thirty studies used brief scoring method of the CDT. The heterogeneity with I^2^ statistic for sensitivity and specificity were 0.86 and 0.83, respectively. The pooled sensitivity and specificity were 0.83 (95% CI = 0.77 to 0.87) and 0.80 (95% CI = 0.74 to 0.85), respectively. The pooled AUC was 88% (95% CI = 85% to 91%). Thirty-five studies used detailed scoring method of the CDT. The heterogeneity with I^2^ statistic for sensitivity and specificity were 0.82 and 0.93, respectively. The pooled sensitivity and specificity were 0.80 (95% CI = 0.76 to 0.83) and 0.81 (95% CI = 0.75 to 0.86), respectively. The pooled AUC was 87% (95% CI = 84% to 90%). No significant difference was found between the diagnostic performance of digital CDT and brief scoring methods (*P* = 0.33) and detailed scoring methods (*P* = 0.35) of paper-and-pencil CDT in the meta-regression model.

### Performance of Other Types of Digital and Paper-and-pencil Drawing Tests

In the screening of MCI, one study used digital ROCF (sensitivity: 0.76, specificity: 0.86, AUC: 85%) and one study used paper-and-pencil ROCF (sensitivity: 0.59, specificity: 0.96, AUC: 77%). In the screening of dementia, two studies used digital pentagon drawing test. The pooled sensitivity and specificity were 0.79 (95% CI = 0.74 to 0.85) and 0.74 (95% CI = 0.68 to 0.78), respectively (Table 2b). Four studies used paper-and-pencil pentagon drawing test. The pooled sensitivity and specificity were 0.85 (95% CI = 0.70 to 0.94) and 73% (95% CI = 0.52 to 0.87), respectively.

**Table 2:** Sensitivity and Specificity of Other types of Digital and Paper-and-Pencil Drawing Tests

Types of Drawing Tests	No. of study	Sensitivity (95% CI)	Specificity 95% CI)	LR +	LR–	DOR	AUC
**a. MCI**							
**Digital Tests**							
– Pentagon Drawing	1	0.71 (0.44–0.99)	0.86 (0.66–1.00)	–	–	–	–
– ROCF	1	0.76	0.86	–	–	–	85%
– Spiral Drawing	1	1.00 (0.72–1.00)	1.00 (0.97–1.00)	–	–	–	–
– House Drawing	1	0.85 (0.61–1.00)	0.94 (0.79–1.00)	–	–	–	–
**Paper-and-pencil Tests**							
– Cube Drawing	1	0.66	0.53	–	–	–	
– ROCF Drawing	1	0.59	0.96	–	–	–	77%
**b. Dementia**							
**Digital Tests**							
– Pentagon Drawing	2	0.79 (0.74–0.85)	0.74 (0.68–0.78)	8.08 (0.42–156)	0.22(0.09–0.54)	41.1 (1.07–157)	–
– Tree Drawing	2	0.88 (0.81–0.93)	0.78 (0.68–0.86)	3.63 (2.07–6.38)	0.17 (0.10–0.27)	22.7 (10.8–47.9)	–
– ROCF	1	0.82	0.91	–	–	–	93%
– House Drawing	1	0.81 (0.69–0.94)	1.00 (0.94–1.00)	–	–	–	–
– Spiral Drawing	1	0.85 (0.73–0.97)	1.00 (0.94–1.00)	–	–	–	–
**Paper-and-pencil Tests**							
– Pentagon Drawing	4	0.85 (0.70–0.94)	0.73 (0.52–0.87)	3.21 (1.82–5.66)	0.20 (0.11–0.36)	16.1 (9.92–26.3)	87% (84%–90%)
– Cube Drawing	1	0.74	0.63	–	–	–	–-

*MCI* Mild Cognitive Impairment, *ROCF* Rey-Osterrieth complex figure, *LR* + Positive likelihood ratio, *LR-* Negative likelihood ration, *DOR* Diagnostic odds ratio, *AUC* Area under the curve, *CI* Confidence interval

### Sensitivity Analyses

Sensitivity analysis showed that the performance of different brief scoring method and detailed scoring method of paper-and-pencil CDT were comparable (Supplementary Table 5).

## Discussion

This systematic review and meta-analysis included 90 studies and compared different types of digital drawing tests and paper-and-pencil drawing tests. The CDT is the most commonly used drawing test. In the screening of MCI, the digital CDT demonstrated better diagnostic performance than the paper-and-pencil CDT. Comparable performance was shown between the digital and paper-and-pencil CDT in the screeing of dementia. The diagnostic performance of other types of digital drawing tests and their paper-and-pencil formats was also comparable. Therefore, digital drawing tests can used as an alternative tool for the screening of MCI and dementia.

There are similarities between the digital CDT and paper-and-pencil CDT. Both methods require participants to draw the clock face as well as the hands of the clock that point to a specific time. The digital CDT uses a digital pen to draw on a tablet instead of drawing on a paper. Previous meta-analyses showed the diagnostic performance of paper-and-pencil CDT is fair in the screening of MCI, no matter the complexity of the scoring system (Ehreke et al., 2010; Pinto & Peters, 2009; Tsoi et al., 2017). This study showed similar results, however, the digital CDT showed better diagnostic performance than paper-and-pencil CDT in the screening of MCI. It may be due to the fact that deterioration of cognitive abilities such as executive function and visuospatial abilities found in patients with MCI are not yet clearly reflected in the final product of the paper drawing. However, decline in cognitive functions may be reflected in the drawing process captured in digital drawing tests (Müller et al., 2017; Garre-Olmo et al., 2017). Among different drawing characteristics, drawing time, pressure acceleration, and velocity are shown to be the behaviour markers for the discrimination of MCI from healthy aging (Garre-Olmo et al., 2017). Müller et al. (2019) further found that drawing time and velocity, such as time-in-air, total time strokes per minute are more sensitive maker than drawing pressure. Müller et al. (2017) suggested that time-in-air was a more sensitive marker than other time factors such as time-on-surface and total time (Muller et al., 2017). Additionally, the digital systems can automatically divide the drawing surface into different segments and sub-regions, and then analyze the strokes and angular differences of the drawing in the calculation of the final score (Davis et al., 2014; Shigemoria, et al., 2015). This combination of visual features and behavioural data can contribute to the identification of patients and enhance the accuracy of the digital CDT (Muller et al., 2019). Therefore, the use of digital CDT can improve the sensitivity and specificity in the screening of MCI. Past work suggests that machine-learning methods can enhance the ability to produce accurate predictive models of the drawing tests to classify MCI and dementia when the models trained on a large amount of data (Davis et al., 2014, Souillard-Mandar et al., 2016; Muller et al., 2017, 2019). Besides digital CDT, some other digital drawing tests have been suggested in the literature. The digital pentagon drawing test (Garre-Olmo et al., 2017; Tsoi et al., 2018) and digital ROCF (Cheah et al., 2019; Kokubo et al., 2018) are adapted from paper-and-pencil versions. The digital tree drawing test and digital house drawing test are adopted new approaches (Robens et al., 2019; Garre-Olmo et al., 2017). The digital tree drawing test and digital house drawing test are free-hand drawing tests which do not require the participants to draw any specific features. Thus, only drawing behaviour are used in the classification of disease.

A comprehensive evaluation of different types of drawing tests for the screening of MCI and dementia is the strength of this study. However, this study has some limitations. First, the comparisons in this study are not head-to-head comparisons, and patients’ engagement and performance on the drawing test is a confounding factor to result interpretation. There is a study of head-to-head comparison between a digital drawing test and a paper-and-pencil drawing test, which showed that digital CDT had a higher diagnostic accuracy than paper-and-pencil CDT (Müller et al., 2017). Second, the number of studies to compare diagnostic performance of drawing tests are limited. The benefits of digital drawing tests may be stronger if we can include more studies in this meta-analysis.

## Conclusions

The current study revealed that digital CDT can enhance the identification of deficits in the screening of MCI. Digital and paper-and-pencil CDT have a comparable performance in the screening of dementia. Other types of drawing tests in digital formats showed comparable to paper-in-pencil formats. Therefore, digital drawing tests can be a potential tool to use as an alternative for the screening of MCI and dementia.

## Supplementary Information

Below is the link to the electronic supplementary material.

Supplementary file1 (DOCX 338 KB)
