# Supplementary material for: Evaluation of Digital Drawing Tests and Paper-and-Pencil Drawing Tests for the Screening of Mild Cognitive Impairment and Dementia: A Systematic Review and Meta-analysis of Diagnostic Studies
Source: Neuropsychol Rev. 2021 Oct 16;32(3):566–76. doi: 10.1007/s11065-021-09523-2 (PMC9381608; doi:10.1007/s11065-021-09523-2)

**Supplemental Table 1. Search Strategies**

| Embase |
| --- |
| 1. (dementia or Cognitive impairment or Alzheimer* or mild cognitive impair* or MCI or Parkinson's disease or dementia with lewy* body or vascular*).af. |
| 2. (drawing test or digital draw* or computerized draw* or digital cognitive test or computerized cognitive test or clock drawing test or CDT or pentagon or cube draw or Rey Osterrieth or ROCF or tree draw or house draw or Spiral or infinity loops).af |
| 3. 1+2 |
| PsycINFO |
| 1. (dementia or Cognitive impairment or Alzheimer* or mild cognitive impair* or MCI or Parkinson's disease or dementia with lewy* body or vascular*).af. |
| 2. (drawing test or digital draw* or computerized draw* or digital cognitive test or computerized cognitive test or clock drawing test or CDT or pentagon or cube draw or Rey Osterrieth or ROCF or tree draw or house draw or Spiral or infinity loops).af |
| 3. 1+2 |
| CINAHL |
| 1. TX (dementia or Cognitive impairment or Alzheimer* or mild cognitive impair* or MCI or Parkinson's disease or dementia with lewy* body or vascular* ) |
| 2. TX (drawing test or digital draw* or computerized draw* or digital cognitive test or computerized cognitive test or clock drawing test or CDT or pentagon or cube draw or Rey Osterrieth or ROCF or tree draw or house draw or Spiral or infinity loops ) |
| 3. 1+2 |
| Medline search via Ebscohost |
| 1. TX (dementia or Cognitive impairment or Alzheimer* or mild cognitive impair* or MCI or Parkinson's disease or dementia with lewy* body or vascular* ) |
| 2. TX (drawing test or digital draw* or computerized draw* or digital cognitive test or computerized cognitive test or clock drawing test or CDT or pentagon or cube draw or Rey Osterrieth or ROCF or tree draw or house draw or Spiral or infinity loops) |
| 3. 1+2 |

**SupplementaryTable 2. Characteristics of Included Studies**

| Study | Region | Age | Male % | Type of Test | No. of Participants | Type of Patients | Quality Score |
| --- | --- | --- | --- | --- | --- | --- | --- |
| Aguilar-Navarro 2018 | Mexico | 75 | NA | Paper-and-pencil CDT – Detailed | 167 | MCI | 8 |
| Alty 2015 | USA, UK | 71 | 68% | Paper-and-pencil Pentagon | 63 | MCI & Dementia | 6 |
| Aprahamian 2010 | Brazil | 78 | 59% | Paper-and-pencil CDT – Brief | 220 | Dementia | 8 |
| Aprahamian 2011 | Brazil | 80 | 29% | Paper-and-pencil CDT – Brief | 106 | Dementia | 8 |
| Beinhoff 2005 | German | 66 | 48% | Paper-and-pencil CDT – Brief | 171 | MCI | 7 |
| Berger 2008 | Germany | 74 | 34% | Paper-and-pencil CDT – Brief | 419 | Dementia | 7 |
| Biundo 2013 | Italy | 64 | 53% | Paper-and-pencil ROCF | 104 | MCI | 8 |
| Brodaty 1997 | Australia | 73 | 32% | Paper-and-pencil CDT – Brief | 56 | Dementia | 6 |
| Cacho 2010 | Spain | 74 | 33% | Paper-and-pencil CDT – Detailed | 163 | MCI | 8 |
| Cahn 1996 | USA | 84 | 40% | Paper-and-pencil CDT – Detailed | 279 | Dementia | 7 |
| Carnero-Pardo 2013 | Spain | 76 | 31% | Paper-and-pencil CDT – Brief | 307 | MCI & Dementia | 8 |
| Cecato 2016 | Brazile | 76 | 36% | Paper-and-pencil Pentagon | 22 | Dementia | 8 |
| Chan 2005 | Hong Kong | 79 | 43% | Paper-and-pencil CDT – Detailed | 85 | Dementia | 8 |
| Charernboon 2017 | Thailand | 70 | 40% | Paper-and-pencil CDT – Brief, Cube | 142 | MCI | 8 |
| Cheah 2019 | Taiwan | 68 | 48% | Digital ROCF | 118 | MCI | 7 |
| Chen 2018 | China | 85 | 35% | Paper-and-pencil CDT – Brief | 361 | MCI | 7 |
| Chiu 2008 | Taiwan | 74 | 59% | Paper-and-pencil CDT – Detailed | 116 | MCI & Dementia | 8 |
| Connor 2005 | USA | 73 | NA | Paper-and-pencil CDT – Detailed | 100 | Dementia | 8 |
| Cormack 2004 | UK | 78 | 88% | Paper-and-pencil Pentagon | 145 | Dementia | 7 |
| Davis 2014 | UK | NA | NA | Digital CDT | 636 | Dementia | 4 |
| Donnelly 2008 | USA | 78 | 99% | Paper-and-pencil CDT – Detailed | 100 | MCI | 8 |
| Ehreke 2011a | Germany | 85 | 4% | Paper-and-pencil CDT – Brief | 428 | MCI | 8 |
| Ehreke 2011b | Germany | 85 | 17% | Paper-and-pencil CDT – Brief | 384 | Dementia | 7 |
| Emek-savas 2018 | Turkey | 71 | NA | Paper-and-pencil CDT – Detailed | 80 | Dementia | 7 |
| Esteban-Santillan 1998 | USA | 72 | 50% | Paper-and-pencil CDT – Detailed | 196 | Dementia | 8 |
| Forti 2010 | Italy | 74 | 33% | Paper-and-pencil CDT – Detailed | 423 | MCI | 8 |
| Fuchs 2012 | Germany | 82 | 39% | Paper-and-pencil CDT – Detailed | 52 | Dementia | 7 |
| Garre-Olmo 2017 | Spain | 64 | NA | Digital CDT, House, Pentagon, Spiral | 102 | MCI | 8 |
| Ghose 2019 | India | 68 | 53% | Paper-and-pencil CDT – Detailed | 114 | MCI | 8 |
| Heinik 2003 | Israel | 78 | 51% | Paper-and-pencil CDT – Detailed | 92 | Dementia | 8 |
| Heymann 2018 | Germany | 69 | 29% | Digital Tree | 140 | MCI & Dementia | 7 |
| Jagar 2003 | UK | 76 | 41% | Paper-and-pencil CDT – Detailed | 231 | MCI | 6 |
| Jorgensen 2014 | Denmark | 77 | 40% | Paper-and-pencil CDT – Brief | 356 | Dementia | 8 |
| Jouk 2012 | Canada | 79 | 49% | Paper-and-pencil CDT – Brief | 104 | Dementia | 7 |
| Kalman 1995 | Hungary | 80 | 53% | Paper-and-pencil CDT – Detailed | 669 | Dementia | 7 |
| Kanchanatawan 2006 | Thailand | NA | NA | Paper-and-pencil CDT – Detailed | 45 | Dementia | 7 |
| Karrasch 2005 | Finland | 72 | 27% | Paper-and-pencil CDT – Brief | 255 | Dementia | 8 |
| Kato 2013 | Japan | 79 | 27% | Paper-and-pencil CDT – Detailed | 56 | Dementia | 7 |
| Kim 2010 | Korea | 71 | NA | Paper-and-pencil CDT – Detailed | 564 | Dementia | 8 |
| Kirby 2001 | Ireland | 80 | NA | Paper-and-pencil CDT – Detailed | 101 | Dementia | 7 |
| Korner 2012 | Denmark | 81 | 38% | Paper-and-pencil CDT – Brief | 247 | Dementia | 8 |
| Ladeira 2009 | Brazil | 70 | NA | Paper-and-pencil CDT – Detailed | 465 | MCI | 8 |
| Lee 2008 | Korea | 72 | 38% | Paper-and-pencil CDT – Detailed | 152 | MCI | 7 |
| Lepeleire 2005 | Belgium | 80 | 53% | Paper-and-pencil CDT – Brief | 364 | Dementia | 6 |
| Lessig 2008 | USA | 78 | 35% | Paper-and-pencil CDT – Detailed | 132 | Dementia | 7 |
| Leung 2005 | Hong Kong | 75 | NA | Paper-and-pencil CDT – Brief | 403 | Dementia | 8 |
| Lin 2003 | Taiwan | 74 | 47% | Paper-and-pencil CDT – Detailed | 211 | Dementia | 8 |
| Lourenço 2008 | Brazil | 74 | 24% | Paper-and-pencil CDT – Brief | 390 | Dementia | 8 |
| Martinelli 2018 | Brazil | 79 | 26% | Paper-and-pencil Pentagon | 176 | Dementia | 8 |
| Matsuoka 2014 | Japan | 80 | 31% | Paper-and-pencil CDT – Detailed | 502 | Dementia | 7 |
| Milian 2012 | Germany | 75 | 37% | Paper-and-pencil CDT – Brief | 299 | Dementia | 7 |
| Moriyama 2016 | Japan | 80 | NA | Paper-and-pencil CDT – Detailed | 70 | MCI & Dementia | 8 |
| Muller 2017 | Germany | 65 | 50% | Digital CDT | 381 | MCI | 6 |
| Muller 2019 | Germany | 65 | 50% | Digital CDT | 157 | MCI | 7 |
| Nair_Anil 2010 | USA | 72 | 46% | Paper-and-pencil CDT – Detailed | 150 | MCI | 7 |
| Nunes 2008 | Brazil | 70 | 30% | Paper-and-pencil CDT – Detailed | 100 | Dementia | 7 |
| Ota 2015 | Japan | 78 | NA | Paper-and-pencil CDT – Detailed, Cube | 132 | Dementia | 8 |
| Parsey 2011 | USA | 71 | 36% | Paper-and-pencil CDT – Detailed | 692 | MCI | 7 |
| Patocskai 2014 | Hungary | 78 | 32% | Paper-and-pencil CDT – Detailed | 75 | Dementia | 8 |
| Powlishta 2002 | USA | 75 | 33% | Paper-and-pencil CDT – Detailed | 188 | Dementia | 8 |
| Rakusa 2018 | Slovenia | 75 | NA | Paper-and-pencil CDT – Brief | 140 | MCI | 8 |
| Ramlall 2013 | South Africa | 77 | 18% | Paper-and-pencil CDT – Detailed | 744 | Dementia | 6 |
| Ravaglia 2003 | Italy | 73 | NA | Paper-and-pencil CDT – Detailed | 190 | Dementia | 8 |
| Ravaglia 2005 | Italy | 76 | 39% | Paper-and-pencil CDT – Detailed | 264 | MCI | 6 |
| Ricci 2016 | Italy | 77 | NA | Paper-and-pencil CDT – Brief | 873 | Dementia | 8 |
| Riedel 2008 | Germany | NA | 63% | Paper-and-pencil CDT – Brief | 177 | Dementia | 8 |
| Robens 2019 | Germany | 68 | 45% | Paper-and-pencil CDT – Brief | 96 | MCI & Dementia | 8 |
| Robert 2003 | France | 78 | 81% | Paper-and-pencil CDT – Brief | 74 | Dementia | 8 |
| Roudsari 2018 | Iran | NA | NA | Paper-and-pencil CDT – Brief | 96 | Dementia | 8 |
| Rubinova 2014 | Czech | 75 | 52% | Paper-and-pencil CDT – Detailed | 142 | MCI | 8 |
| Russo 2014 | Argentina | NA | 30% | Paper-and-pencil CDT – Detailed | 80 | MCI & Dementia | 6 |
| Scanlan 2002 | USA | NA | NA | Paper-and-pencil CDT – Detailed | 369 | Dementia | 6 |
| Schmidtke 2006 | Germany | 76 | 34% | CDT-brief | 337 | Dementia | 6 |
| Seigerschmidt 2002 | Germany | 75 | 37% | Paper-and-pencil CDT – Detailed | 190 | MCI | 5 |
| Shigemori 2015 | Japan | NA | NA | Digital CDT | 127 | MCI | 7 |
| Storey 2001 | Australia | 79 | 29% | Paper-and-pencil CDT – Brief | 93 | Dementia | 8 |
| Storey 2002 | Australia | 80 | 33% | Paper-and-pencil CDT – Brief | 150 | Dementia | 7 |
| Sunderland 1989 | USA | 68 | NA | Paper-and-pencil CDT – Detailed | 50 | Dementia | 7 |
| Todd 1995 | USA | 58 | NA | Paper-and-pencil CDT – Detailed | 215 | Dementia | 8 |
| Trenkle 2007 | USA | 75 | 53% | Paper-and-pencil CDT – Detailed | 465 | Dementia | 6 |
| Tsoi 2018 | Hong Kong | 80 | NA | Digital Pentagon | 473 | Dementia | 8 |
| Van de Burg 2004 | Belgium | 65 | NA | Paper-and-pencil CDT – Brief | 187 | Dementia | 8 |
| Vyhnalek 2017 | Czech Republic | 76 | 57% | Paper-and-pencil CDT – Detailed | 76 | MCI | 7 |
| Watson 1993 | USA | 76 | 19% | Paper-and-pencil CDT – Detailed | 224 | Dementia | 7 |
| Wiechmann 2010 | USA | 75 | 30% | Paper-and-pencil CDT – Brief | 277 | Dementia | 6 |
| Wolf-Klein 1989 | USA | 79 | NA | Paper-and-pencil CDT – Brief | 219 | Dementia | 7 |
| Yamamoto 2004 | Japan | 75 | 44% | Paper-and-pencil CDT – Detailed | 2015 | MCI | 8 |
| Yang 2016 | China | 84 | 36% | Paper-and-pencil CDT – Brief | 148 | Dementia | 8 |
| Yap 2007 | Singapore | 78 | 38% | Paper-and-pencil CDT – Detailed | 160 | Dementia | 7 |
| Zhou 2008 | China | 66 | 70% | Paper-and-pencil CDT – Detailed | 167 | MCI | 8 |

Abbveiations: CDT, Clock Drawing Test, MCI, Mild Cognitive Impariment; NA, Not Avaliable

**Supplementary Table 3. Risk of Bias of Included Studies**

| Study & Year | 1. Patient selection | 2. Execution of the index test | 3. Execution of the ref. standard | 4. Flow and timing |
| --- | --- | --- | --- | --- |
| Aguilar-Navarro 2018 | L | L | L | L |
| Alty 2015 | L | H | H | L |
| Aprahamian 2010 | L | L | L | L |
| Aprahamian 2011 | L | U | L | L |
| Beinhoff_2005 | L | U | L | L |
| Berger 2008 | L | L | L | L |
| Biundo 2013 | L | U | L | L |
| Brodaty 1997 | L | U | U | L |
| Cacho 2010 | L | U | U | L |
| Cahn 1996 | L | L | L | L |
| Carnero-Pardo 2013 | L | L | L | L |
| Cecato 2016 | U | U | L | U |
| Chan_2005 | L | U | L | U |
| Charernboon 2017 | L | U | L | U |
| Cheah 2019 | H | L | H | L |
| Chen 2018 | L | L | U | U |
| Chiu_ 2008 | L | U | L | L |
| Connor2005 | L | L | L | L |
| Cormack 2004 | L | L | L | U |
| Davis 2014 | H | U | L | H |
| Donnelly 2008 | L | L | L | L |
| Ehreke_2011a | L | L | L | L |
| Ehreke 2011b | L | L | L | L |
| Emek-savas 2018 | L | L | L | L |
| Esteban-Santillan 1998 | H | L | L | L |
| Forti 2010 | L | U | L | L |
| Fuchs 2012 | L | U | L | L |
| Garre-Olmo 2017 | L | L | L | L |
| Ghose_2019 | L | U | L | L |
| Heinik_2003 | L | L | L | U |
| Heymann_2018 | L | L | L | L |
| Jagar_2003 | L | U | L | L |
| Jorgensen 2014 | L | L | H | U |
| Jouk 2012 | L | L | L | L |
| Kalman 1995 | U | L | L | L |
| Kanchanatawan 2006 | U | U | L | L |
| Karrasch 2005 | U | U | L | U |
| Kato 2013 | L | L | L | L |
| Kim 2010 | U | L | L | L |
| Kirby 2001 | U | L | L | U |
| Korner 2012 | U | L | L | U |
| Ladeira_2009 | L | U | U | L |
| Lee 2008 | L | L | L | L |
| Lepeleire 2005 | L | U | U | U |
| Lessig 2008 | L | U | L | U |
| Leung 2005 | L | L | L | L |
| Lin 2003 | U | L | L | L |
| Lourenço 2008 | U | L | L | L |
| Martinelli 2018 | U | U | L | L |
| Matsuoka 2014 | L | L | L | L |
| Milian 2012 | L | U | L | L |
| Moriyama 2016 | U | L | L | L |
| Muller_2017 | U | L | L | L |
| Muller_2019 | U | L | L | U |
| Nair_Anil 2010 | L | L | L | L |
| Nunes 2008 | U | L | L | U |
| Ota 2015 | H | L | L | L |
| Parsey 2011 | U | L | L | L |
| Patocskai 2014 | U | L | L | U |
| Powlishta 2002 | U | L | L | H |
| Rakusa 2018 | L | L | L | L |
| Ramlall 2013 | L | L | L | L |
| Ravaglia 2003 | L | L | U | L |
| Ravaglia 2005 | L | L | L | L |
| Ricci 2016 | L | L | U | U |
| Riedel 2008 | L | L | L | L |
| Robens 2019 | L | L | U | L |
| Robert 2003 | L | L | U | L |
| Roudsari 2018 | L | L | L | L |
| Rubinova 2014 | L | L | U | U |
| Russo 2014 | L | L | L | L |
| Scanlan 2002 | L | L | L | U |
| Schmidtke 2006 | L | L | L | L |
| Seigerschmidt 2002 | L | L | U | U |
| Shigemori 2015 | H | L | U | H |
| Storey 2001 | L | L | U | U |
| Storey 2002 | U | U | L | L |
| Sunderland 1989 | U | L | L | L |
| Todd 1995 | L | L | L | L |
| Trenkle 2007 | L | L | L | L |
| Tsoi 2018 | L | L | U | L |
| Van de Burg 2004 | L | L | L | L |
| Vyhnalek_2017 | L | L | L | L |
| Watson 1993 | L | L | L | L |
| Wiechmann 2010 | H | L | L | H |
| Wolf-Klein 1989 | L | U | L | U |
| Yamamoto 2004 | L | L | L | L |
| Yang 2016 | L | U | L | L |
| Yap 2007 | L | L | L | L |
| Zhou 2008 | U | L | L | L |

Abbreviations: L, Low riks; H, High risk; U, Uncertain risk

**Supplementary Table 4. Description of Results of Included Studies**

| Study & Year | Patient Type | Type of test | True Positive | False Negative | False Negative | True Positive |
| --- | --- | --- | --- | --- | --- | --- |
| Aguilar-Navarro 2018 | MCI | Paper-and-pencil CDT – Detailed | 21 | 17 | 31 | 41 |
| Aguilar-Navarro_2018 | Dementia | Paper-and-pencil CDT – Detailed | 51 | 3 | 6 | 55 |
| Alty2015 | Dementia | Pentagon | 8 | 5 | 23 | 27 |
| Aprahamian 2010 | Dementia | Paper-and-pencil CDT – Brief | 104 | 33 | 17 | 66 |
| Aprahamian 2011 | Dementia | Paper-and-pencil CDT – Brief | 44 | 7 | 22 | 33 |
| Beinhoff_2005 | MCI | Paper-and-pencil CDT – Brief | 19 | 23 | 29 | 34 |
| Beinhoff_2005 | Dementia | Paper-and-pencil CDT – Brief | 47 | 3 | 19 | 54 |
| Berger 2008 | Dementia | Paper-and-pencil CDT – Brief | 301 | 56 | 33 | 72 |
| Biundo_2013 | MCI | ROCF | 20 | 2 | 14 | 53 |
| Brodaty_1997 | Dementia | Paper-and-pencil CDT – Brief | 24 | 1 | 4 | 27 |
| Cacho 2010 | MCI | Paper-and-pencil CDT – Detailed | 16 | 20 | 5 | 46 |
| Cacho 2010 | Dementia | Paper-and-pencil CDT – Detailed | 48 | 2 | 18 | 64 |
| Cahn_1996 | Dementia | Paper-and-pencil CDT – Detailed | 35 | 66 | 7 | 171 |
| Cecato_2016 | Dementia | Pentagon | 14 | 8 | 0 | 14 |
| Chan_2005 | Dementia | Paper-and-pencil CDT – Detailed | 46 | 18 | 5 | 16 |
| Charernboon_2017 | MCI | Cube | 23 | 28 | 12 | 32 |
| Charernboon_2017 | MCI | Paper-and-pencil CDT – Brief | 20 | 18 | 15 | 42 |
| Cheah_2019 | MCI | Digital ROCF | 45 | 8 | 14 | 51 |
| chen_2018 | MCI | Paper-and-pencil CDT – Brief | 97 | 39 | 21 | 94 |
| chen_2018 | Dementia | Paper-and-pencil CDT – Brief | 102 | 39 | 8 | 94 |
| Chiu_2008 | mci | Paper-and-pencil CDT – Detailed | 25 | 17 | 9 | 23 |
| Chiu_2008 | Dementia | Paper-and-pencil CDT – Detailed | 24 | 6 | 18 | 34 |
| Connor_2005 | Dementia | Paper-and-pencil CDT – Detailed | 37 | 6 | 13 | 44 |
| Cormack_2004 | Dementia | Pentagon | 37 | 13 | 13 | 68 |
| Davis 2014 | Dementia | Digital CDT | 48 | 84 | 28 | 476 |
| Donnelly_2008 | MCI | Paper-and-pencil CDT – Detailed | 17 | 45 | 3 | 35 |
| Ehreke 2011a | Dementia | Paper-and-pencil CDT – Brief | 19 | 125 | 9 | 231 |
| Ehreke_2011b | MCI | Paper-and-pencil CDT – Brief | 44 | 155 | 14 | 215 |
| Emek-savas_2018 | Dementia | Paper-and-pencil CDT – Detailed | 97 | 25 | 11 | 83 |
| Emek-savas_2018 | MCI | Paper-and-pencil CDT – Detailed | 101 | 25 | 24 | 100 |
| Esteban-Santillan 1998 | Dementia | Paper-and-pencil CDT – Detailed | 38 | 23 | 3 | 16 |
| Forti 2010 | MCI | Paper-and-pencil CDT – Detailed | 88 | 23 | 44 | 41 |
| Fuchs 2012 | Dementia | Paper-and-pencil CDT – Detailed | 19 | 66 | 2 | 336 |
| Garre-Olmo_2017 | MCI | Digital CDT | 11 | 0 | 1 | 17 |
| Garre-Olmo_2017 | Dementia | Digital CDT | 29 | 4 | 6 | 13 |
| Garre-Olmo_2017 | Dementia | Digital Pentagon | 26 | 0 | 3 | 23 |
| Garre-Olmo_2017 | Dementia | Digital Spiral | 25 | 2 | 4 | 21 |
| Garre-Olmo_2017 | Dementia | Digital house | 24 | 0 | 5 | 23 |
| Ghose_2019 | MCI | Paper-and-pencil CDT – Detailed | 32 | 22 | 8 | 40 |
| Heinik_2003 | Dementia | Paper-and-pencil CDT – Detailed | 75 | 3 | 13 | 23 |
| Heymann_2018 | Dementia | Digital Tree | 61 | 8 | 7 | 16 |
| Heymann_2018 | Dementia | Digital CDT | 50 | 0 | 18 | 24 |
| Jagar_2003 | MCI | Paper-and-pencil CDT – Detailed | 22 | 28 | 7 | 23 |
| Jagar_2003 | Dementia | Paper-and-pencil CDT – Detailed | 54 | 15 | 6 | 36 |
| Jorgensen_2014 | Dementia | Paper-and-pencil CDT – Brief | 88 | 19 | 48 | 76 |
| Jouk_2012 | Dementia | Paper-and-pencil CDT – Brief | 65 | 88 | 15 | 188 |
| Kalman 1995 | Dementia | Paper-and-pencil CDT – Detailed | 35 | 5 | 10 | 54 |
| Kanchanatawan 2006 | Dementia | Paper-and-pencil CDT – Detailed | 22 | 167 | 3 | 477 |
| Karrasch_2005 | Dementia | Paper-and-pencil CDT – Brief | 5 | 2 | 10 | 13 |
| Kato 2013 | Dementia | Paper-and-pencil CDT – Detailed | 121 | 6 | 25 | 43 |
| Kato 2013 | MCI | Paper-and-pencil CDT – Detailed | 30 | 6 | 30 | 43 |
| Kim_2010 | Dementia | Paper-and-pencil CDT – Detailed | 16 | 3 | 12 | 25 |
| Kirby 2001 | Dementia | Paper-and-pencil CDT – Detailed | 31 | 99 | 10 | 424 |
| Korner 2012 | Dementia | Paper-and-pencil CDT – Brief | 61 | 4 | 11 | 25 |
| Ladeira_2009 | MCI | Paper-and-pencil CDT – Detailed | 25 | 10 | 58 | 73 |
| Ladeira_2009 | Dementia | Paper-and-pencil CDT – Detailed | 49 | 10 | 32 | 73 |
| Lee 2008 | MCI | Paper-and-pencil CDT – Detailed | 91 | 41 | 133 | 200 |
| Lee 2008 | MCI | Paper-and-pencil CDT – Brief | 96 | 35 | 128 | 206 |
| lepeleire_2005 | Dementia | Paper-and-pencil CDT – Brief | 34 | 17 | 17 | 85 |
| Lessig_2008 | Dementia | Paper-and-pencil CDT – Detailed | 199 | 40 | 27 | 98 |
| Leung 2005 | Dementia | Paper-and-pencil CDT – Brief | 52 | 24 | 14 | 42 |
| Lin_2003 | Dementia | Paper-and-pencil CDT – Detailed | 96 | 66 | 48 | 193 |
| Lourenço 2008 | Dementia | Paper-and-pencil CDT – Brief | 44 | 61 | 24 | 82 |
| Martinelli 2018 | Dementia | Pentagon | 253 | 67 | 13 | 57 |
| Matsuoka 2014 | Dementia | Paper-and-pencil CDT – Detailed | 80 | 7 | 51 | 38 |
| Matsuoka 2014 | Dementia | Paper-and-pencil CDT – Brief | 80 | 7 | 51 | 38 |
| Milian 2012 | Dementia | Paper-and-pencil CDT – Brief | 342 | 2 | 96 | 62 |
| Muller_2017 | MCI | Digital CDT | 20 | 6 | 4 | 14 |
| Muller_2017 | MCI | Paper-and-pencil CDT – Brief | 20 | 6 | 4 | 14 |
| Muller_2017 | Dementia | Digital CDT | 17 | 3 | 3 | 17 |
| Muller_2019 | MCI | Digital CDT | 118 | 31 | 20 | 106 |
| Muller_2019 | Dementia | Digital CDT | 97 | 8 | 9 | 129 |
| Nair_Anil 2010 | MCI | Paper-and-pencil CDT – Detailed | 41 | 27 | 9 | 23 |
| Nair_Anil 2010 | Dementia | Paper-and-pencil CDT – Detailed | 42 | 12 | 8 | 38 |
| Nunes 2008 | Dementia | Paper-and-pencil CDT – Detailed | 25 | 21 | 5 | 41 |
| Nunes 2008 | MCI | Paper-and-pencil CDT – Detailed | 34 | 21 | 31 | 41 |
| Ota 2015 | Dementia | Paper-and-pencil CDT – Detailed | 54 | 16 | 16 | 14 |
| Ota 2015 | Dementia | Cube | 52 | 15 | 18 | 15 |
| Parsey 2011 | MCI | Paper-and-pencil CDT – Detailed | 13 | 8 | 20 | 58 |
| Patocskai_2014 | Dementia | Paper-and-pencil CDT – Detailed | 229 | 140 | 34 | 151 |
| Powlishta 2002 | Dementia | Paper-and-pencil CDT – Detailed | 45 | 3 | 15 | 12 |
| Powlishta 2002 | Dementia | Paper-and-pencil CDT – Brief | 42 | 2 | 18 | 13 |
| Rakusa 2018 | MCI | Paper-and-pencil CDT – Brief | 34 | 4 | 15 | 39 |
| Rakusa 2018 | Dementia | Paper-and-pencil CDT – Brief | 48 | 4 | 6 | 39 |
| Ramlall_2013 | Dementia | Paper-and-pencil CDT – Detailed | 5 | 10 | 6 | 81 |
| Ravaglia 2003 | Dementia | Paper-and-pencil CDT – Detailed | 34 | 28 | 9 | 673 |
| Ravaglia 2003 | Dementia | Paper-and-pencil CDT – Brief | 37 | 49 | 6 | 652 |
| Ravaglia_2005 | MCI | Paper-and-pencil CDT – Detailed | 23 | 8 | 34 | 47 |
| Ravaglia_2005 | MCI | Paper-and-pencil CDT – Brief | 4 | 6 | 14 | 49 |
| Ricci 2016 | Dementia | Paper-and-pencil CDT – Brief | 167 | 34 | 16 | 304 |
| Ricci 2016 | MCI | Paper-and-pencil CDT – Brief | 150 | 54 | 48 | 284 |
| Riedel_2008 | Dementia | Paper-and-pencil CDT – Brief | 178 | 191 | 72 | 432 |
| Robens_2019 | MCI | Digital Tree | 36 | 11 | 28 | 56 |
| Robens_2019 | Dementia | Digital Tree | 48 | 12 | 8 | 55 |
| Robert_2003 | Dementia | Paper-and-pencil CDT – Brief | 27 | 6 | 22 | 41 |
| Roudsari_2018 | Dementia | Paper-and-pencil CDT – Brief | 36 | 9 | 4 | 25 |
| Rubinova 2014 | MCI | Paper-and-pencil CDT – Detailed | 30 | 18 | 18 | 30 |
| Russo 2014 | MCI | Paper-and-pencil CDT – Detailed | 67 | 1 | 50 | 29 |
| Russo 2014 | Dementia | Paper-and-pencil CDT – Detailed | 36 | 1 | 20 | 29 |
| Scanlan 2002 | Dementia | Paper-and-pencil CDT – Detailed | 33 | 2 | 10 | 23 |
| Schmidtke_2006 | Dementia | Paper-and-pencil CDT – Brief | 113 | 14 | 17 | 50 |
| Seigerschmidt 2002 | MCI | Paper-and-pencil CDT – Detailed | 66 | 48 | 33 | 91 |
| Shigemori_2015 | MCI | Digital CDT | 2 | 2 | 1 | 90 |
| Shigemori_2015 | Dementia | Digital CDT | 94 | 20 | 6 | 90 |
| Storey 2001 | Dementia | Paper-and-pencil CDT – Brief | 67 | 25 | 5 | 30 |
| Storey 2002 | Dementia | Paper-and-pencil CDT – Brief | 38 | 18 | 11 | 26 |
| Sunderland_1989 | Dementia | Paper-and-pencil CDT – Detailed | 52 | 3 | 15 | 80 |
| Todd_1995 | Dementia | Paper-and-pencil CDT – Detailed | 22 | 4 | 1 | 23 |
| Trenkle_2007 | Dementia | Paper-and-pencil CDT – Detailed | 30 | 43 | 21 | 27 |
| Tsoi_2018 | Dementia | Digital Pentagon | 151 | 78 | 43 | 193 |
| Van de Burg 2004 | Dementia | Paper-and-pencil CDT – Brief | 372 | 50 | 15 | 36 |
| Vyhnalek_2017 | MCI | Paper-and-pencil CDT – Detailed | 34 | 33 | 15 | 55 |
| Vyhnalek_2017 | Dementia | Paper-and-pencil CDT – Detailed | 46 | 33 | 4 | 55 |
| Watson_1993 | Dementia | Paper-and-pencil CDT – Detailed | 35 | 6 | 5 | 30 |
| Wiechmann 2010 | Dementia | Paper-and-pencil CDT – Brief | 200 | 7 | 0 | 17 |
| Wolf-Klein_1989 | Dementia | Paper-and-pencil CDT – Brief | 105 | 14 | 16 | 177 |
| Yamamoto_2004 | MCI | Paper-and-pencil CDT – Detailed | 36 | 10 | 12 | 31 |
| Yang_2016 | Dementia | Paper-and-pencil CDT – Brief | 404 | 286 | 40 | 1285 |
| Yap_2007 | Dementia | Paper-and-pencil CDT – Detailed | 55 | 15 | 18 | 60 |
| Zhou 2008 | MCI | Paper-and-pencil CDT – Detailed | 55 | 17 | 25 | 63 |

Abbveiations: CDT, Clock Drawing Test, MCI, Mild Cognitive Impariment;

**Supplementary Table 5. Sensitivity Anlyais of Sensitivity and Specificity of Commonly Used Scoring Methods of Paper-and-Pencil Clock Drawing Tests**

| **Paper-and-Pencil CDT** | **No. of study** | **Sensitivity**  **(95% CI)** | **Specificity**  **95% CI)** | **LR+** | **LR-** | **DOR** | **AUC** |
| --- | --- | --- | --- | --- | --- | --- | --- |
| **Brief Scoring** |  |  |  |  |  |  |  |
| 5-point | 4 | 0.78 (0.64 – 0.88) | 0.83 (0.71 – 0.90) | 4.47 (2.43 – 8.22) | 0.26 (0.14 – 0.48) | 16.9 95.68 – 50.3) | 88% (84%-90%) |
| 6-point | 22 | 0.80 (0.74 – 0.85) | 0.75 (0.67 – 0.82) | 3.27 (2.46 – 4.35) | 0.26 (0.20 – 0.34) | 12.6 (8.00 – 19.8) | 85% (82%-88%) |
| **Detailed Scoring** |  |  |  |  |  |  |  |
| 10-point | 23 | 0.79 (0.75 – 0.83) | 0.80 (0.73 – 0.86) | 4.01 (2.92 – 5.51) | 0.26 (0.21 – 0.31) | 15.6 (10.5 – 23.2) | 86% (82%-88%) |
| 14-point | 4 | 0.75 (0.46 – 0.92) | 0.81 (0.56 – 0.94) | 4.04 (1.53 – 10.7) | 0.30 (0.12 – 0.76) | 13.3 (2.94 – 60.4) | 85% (82%-88%) |
| 15-point | 7 | 0.75 (0.65 – 0.82) | 0.71 (0.58 – 0.81) | 0.36 (0.23 – 0.55) | 0.36 (0.23 – 0.55) | 7.31 (3.11 – 17.2) | 79% (76%-83%) |

**Supplementary Figure 1. HSROC for the Pooled Sensitivity and Specificity of Digital and Paper-and Pencil Clock Drawing Test for the Screening of MCI**


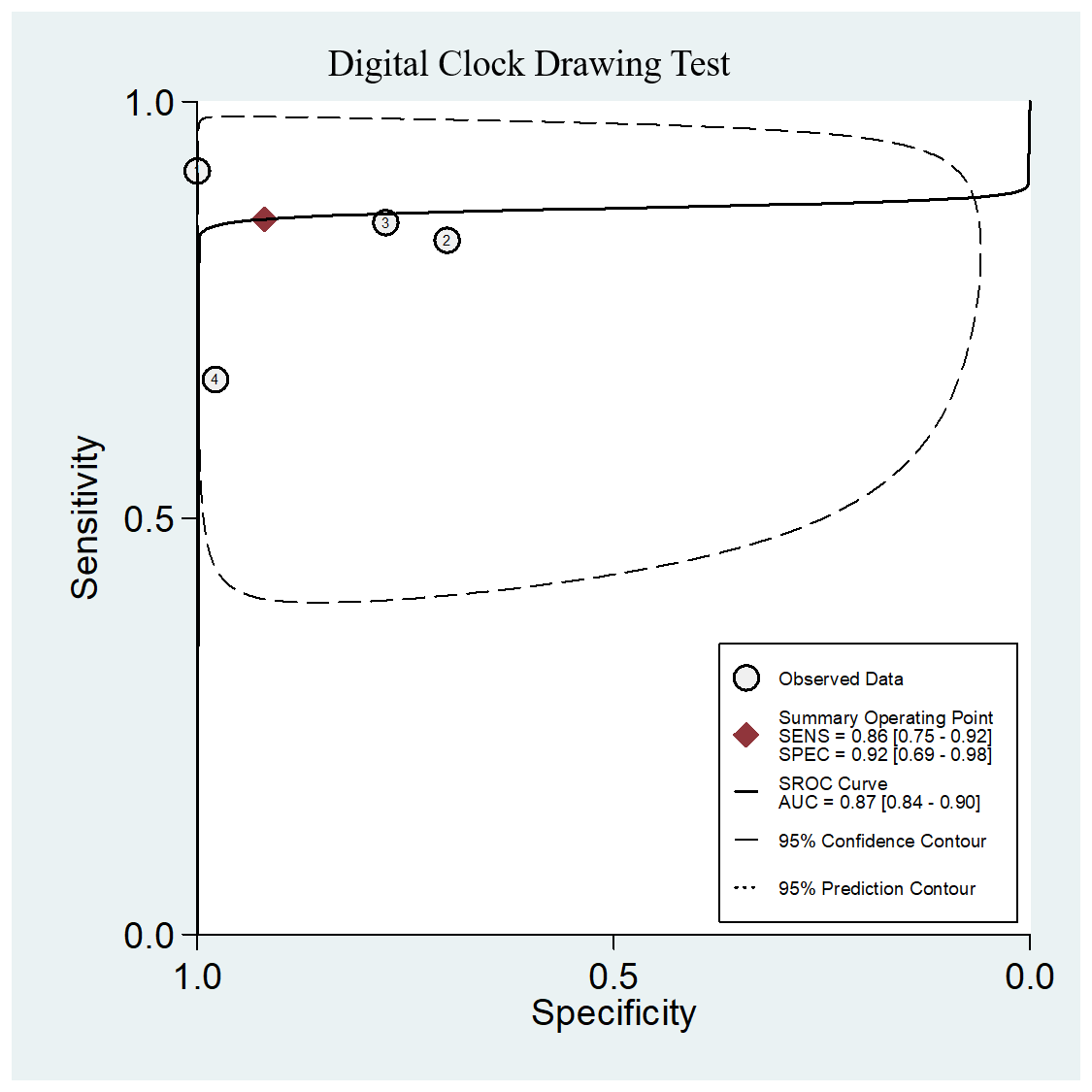


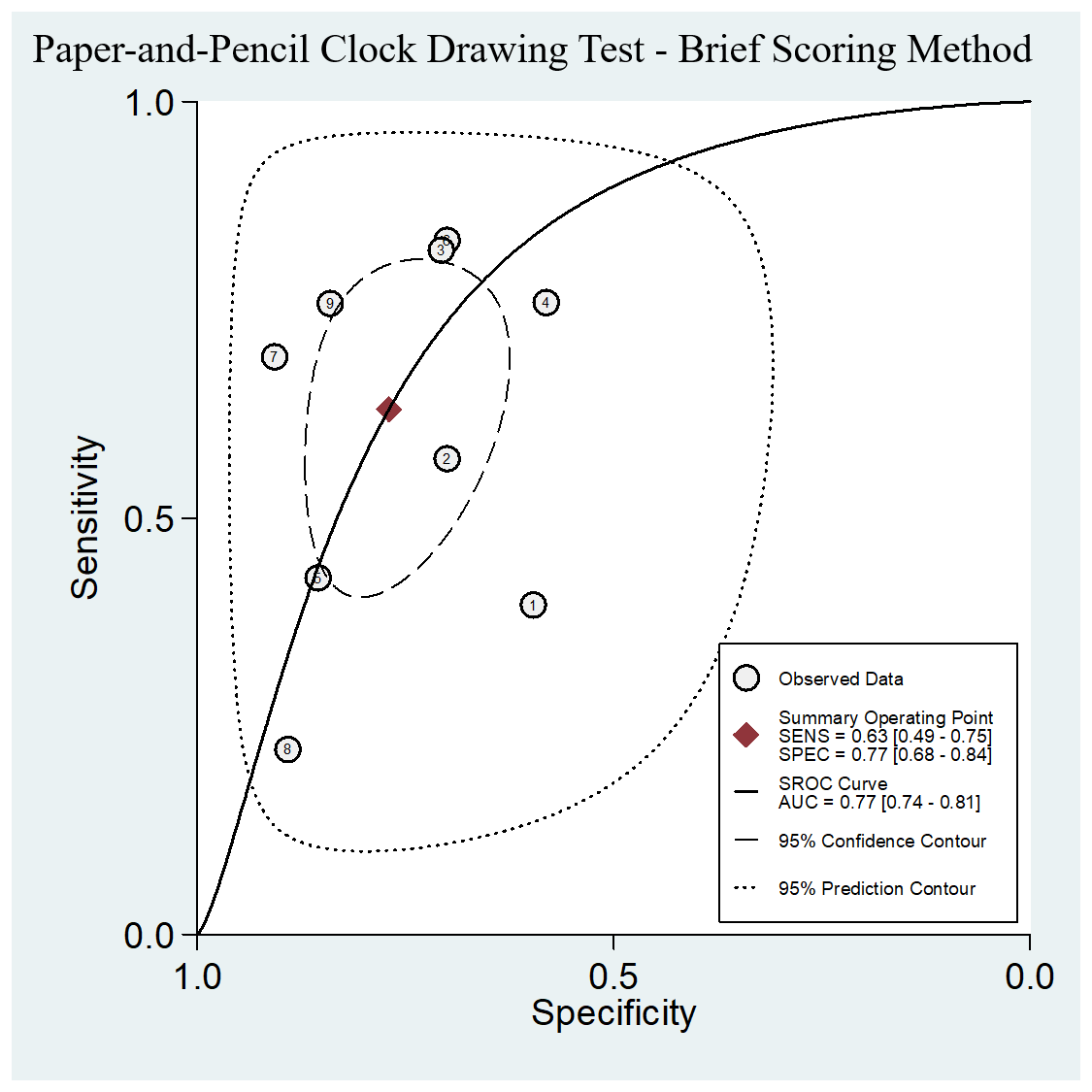


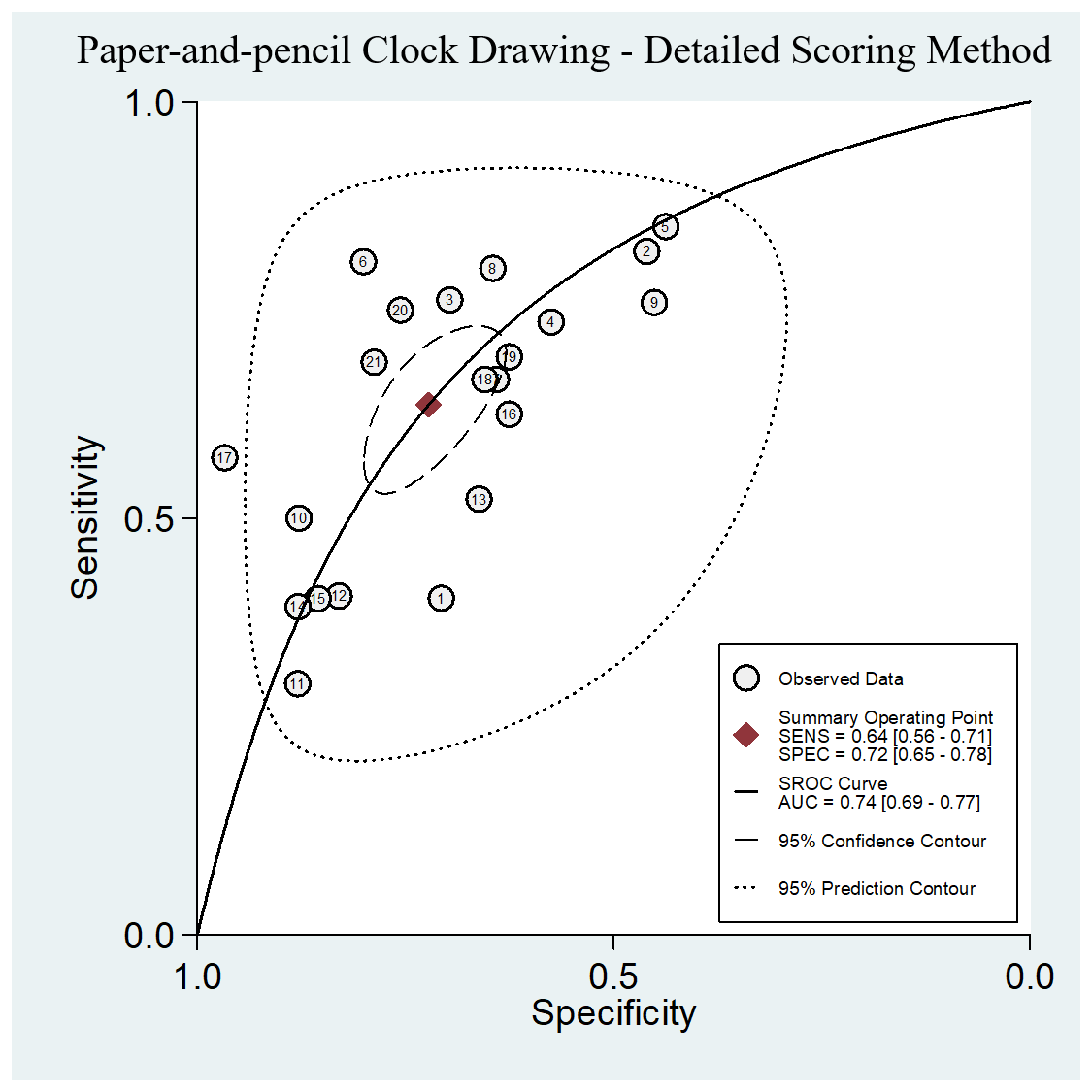


**Supplementary Figure 2. Funnel Plots of Publication Bias**

a. Funnel Plot of Digital CDT for the screening of MCI

b. Funnel Plot of Paper-and-pencil CDT – Brief scoring method for the screening of MCI

c. Funnel Plot of Paper-and-pencil CDT – Detailed scoring method for the screening of MCI

d. Funnel Plot of Digital CDT for the screening of dementia

e. Funnel Plot of Paper-and-pencil CDT – Brief scoring method for the screening of dementia

f. Funnel Plot of Paper-and-pencil CDT – Detailed scoring method for the screening of dementia

**Supplementary Figure 3. HSROC for the Pooled Sensitivity and Specificity of Digital and Paper-and Pencil Clock Drawing Test for the Screening of Dementia**


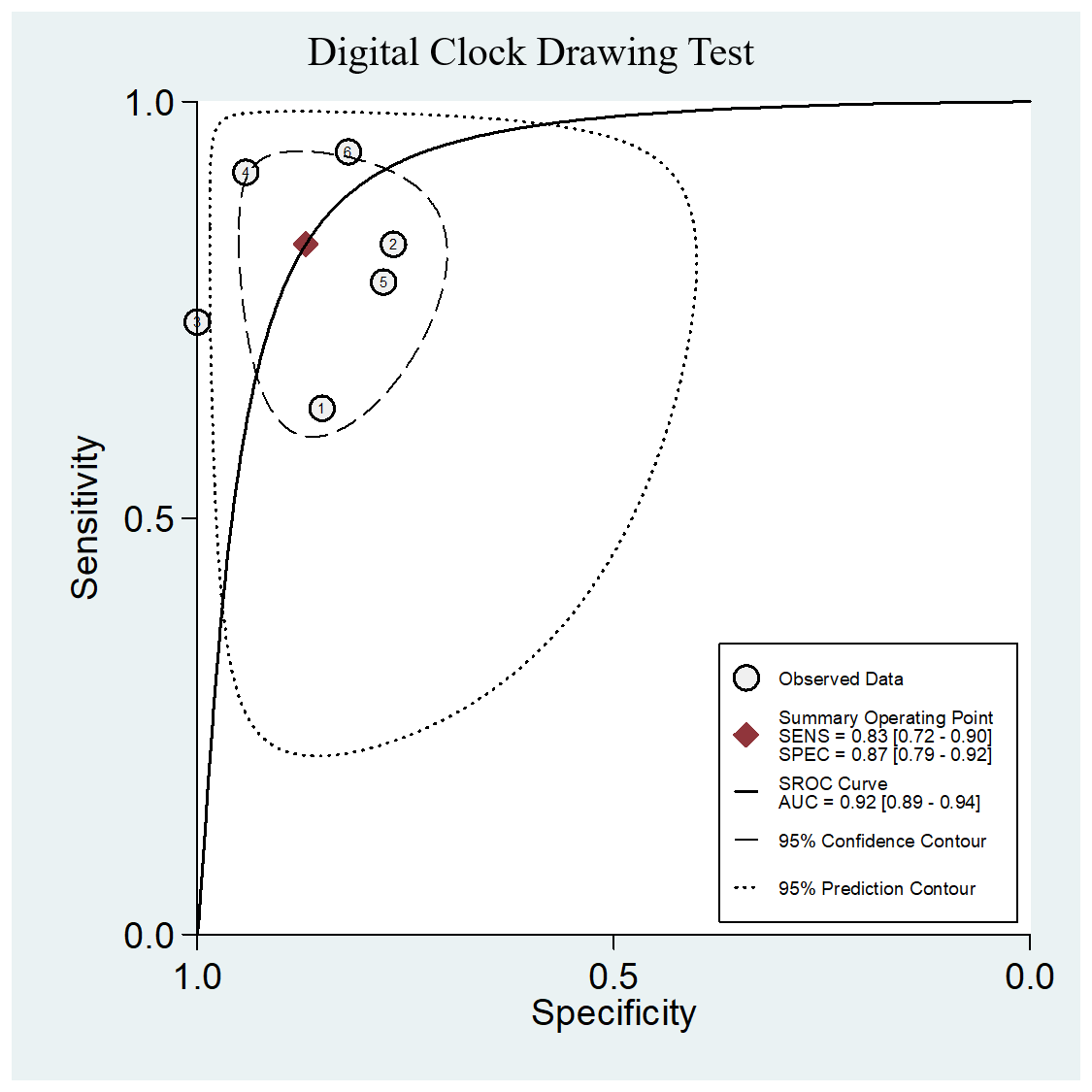


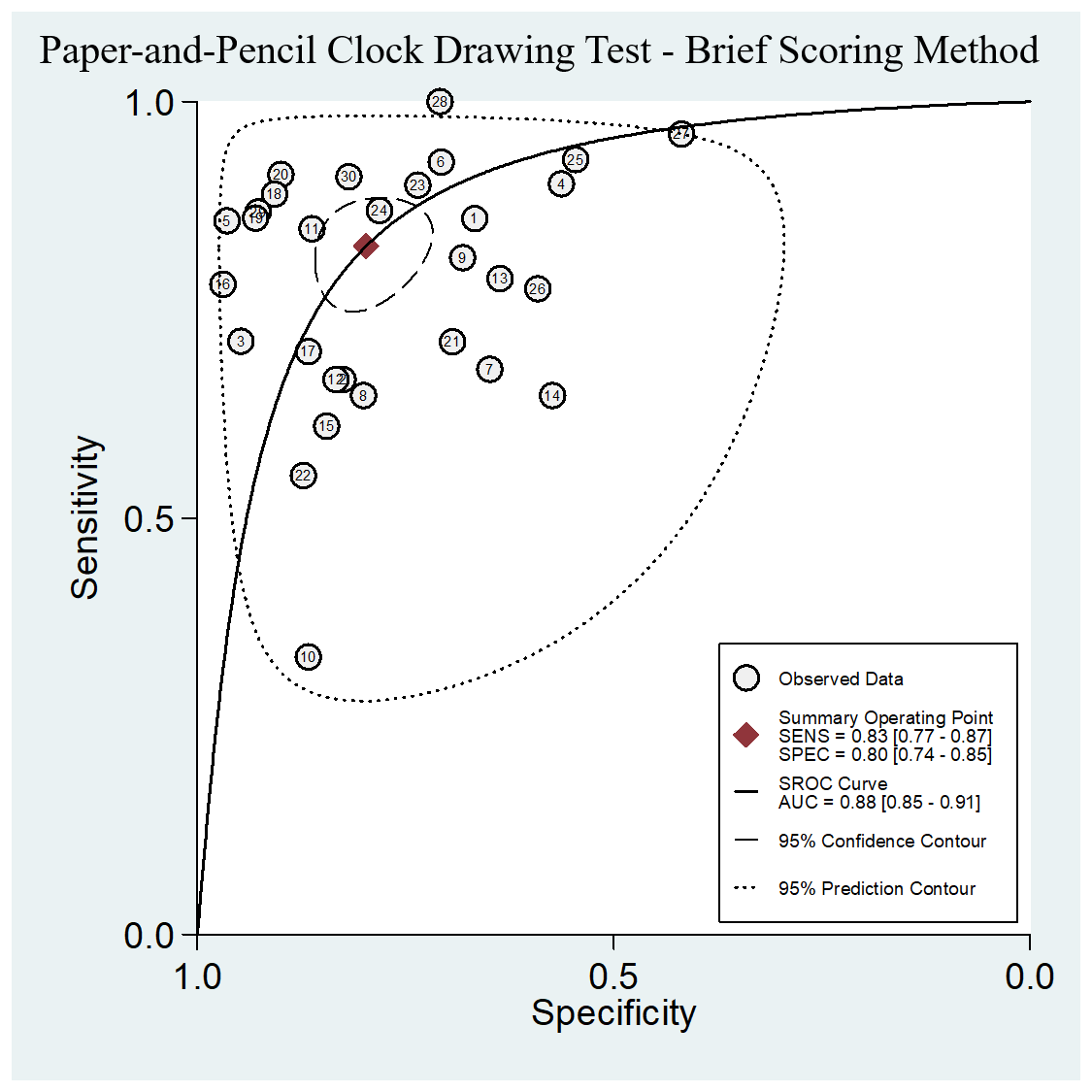

Supplement: Supplementary file 1 — Supplementary file1 (DOCX 338 KB) [file 11065_2021_9523_MOESM1_ESM.docx]
